# Taohong Siwu decoction for femoral head necrosis: A protocol for systematic review

**DOI:** 10.1097/MD.0000000000019368

**Published:** 2020-03-27

**Authors:** Guoming Chen, Yaying Xie, Yunyun Liu, Shanmi Jin, Ziyin Chen, Peng Zhang, Peiyu Shi, Junxia Zhu, Jieyi Deng, Haorui Liang, Chi Zhou

**Affiliations:** a Guangzhou University of Chinese Medicine; b Department of Orthopedics, First Affiliated Hospital of Guangzhou University of Chinese Medicine, Guangzhou, China.

**Keywords:** femoral head necrosis, meta-analysis protocol, systematic review, Taohong Siwu decoction

## Abstract

**Backgrounds:**

Femoral head necrosis is one of the most common orthopedic diseases which can be diagnosed in all ages with different reasons. Taohong Siwu decoction (TSD) has been widely used in the treatment of femoral head necrosis. However, as far as we know, there is still a lack of supporting evidence regarding the efficacy of TSD for femoral head necrosis. Therefore, this protocol aims to evaluate the effectiveness and safety of TSD for femoral head necrosis.

**Methods:**

Eight electronic databases, including PubMed, the Cochrane Central Register of Controlled Trials, EMBASE, Web of Science, Chinese Biomedical Literature Database, China National Knowledge Infrastructure, Technology Periodical database, (Chinese Scientific Journal Database) and Wanfang Database will be searched from the time when the respective databases were established to January 2020. Randomized controlled trials of TSD in the treatment of femoral head necrosis will be collected. After evaluating the quality of methodology and extracting valid data, the final meta-analysis will be carried out with software Revman 5.3.

**Ethics and dissemination:**

The results of this systematic review will offer implications of the use of TSD treatment for Femoral Head Necrosis. It uses aggregated published data instead of individual patient data and does not require an ethical board review and approval. The findings will be published in a peer-reviewed journal and disseminated in conference presentations.

**Results:**

The results of this study will offer implications of the use of TSD treatment for FHN with this meta-analysis.

**Conclusion:**

The conclusion of this study will provide recent evidence to assess whether TSD is effective and safe in the treatment of FHN.

## Abstract 2

Supplemental Digital Content is available in the text

## 1 Introduction

Femoral head necrosis (FHN) is a painful bone disease which can be divided into 2 kinds: traumatic FHN and nontraumatic FHN.^[1]^ Although its nosogenesis is not completely clarified, some potential mechanisms of FHN such as osteocyte apoptosis, cell differentiation disorder, and angiogenesis hindrance are raised by scientists.^[2]^ People affected by FHN are mainly between 20 and 40 years old.^[3]^ And those affected by FHN may probably suffer from long-term symptoms.^[2]^ In the United States, about 20,000 to 30,000 patients are diagnosed with FHN every year.^[4]^

The treatments of FHN include nonsurgical treatment and surgical treatment.^[4]^ There are several options of nonoperative management: limiting weight with a walker, activity modification, and physical therapy to reduce the force on the hip joint and lipid-reducing agents, bisphosphonates, and hyperbaric oxygen.^[5,6]^ However, the treatments listed above have minimal effect after subchondral collapse of femoral head has appeared.^[6]^ Among various kinds of treatment of FHN, core decompression is the most common therapy to treat pre-collapse FHN which can relieve pain on the early stage of bone illness.^[3,7]^ It is a prophylactic surgery which works by drilling the necrotic cancellous bone in the femoral head and taking it from a lateral femoral cortical entry point.^[3,6,8]^ Besides, mesenchymal stem cells also play a significant role in the therapies of FHN.^[3]^ When patients with FHN reach a certain stage of disease development, total hip arthroplasty may be essential.^[6]^

Traditional Chinese medicine (TCM) is the crystallization of ancient Chinese life's wisdom. It greatly complements and enriches the content of modern medicine. These days, related research has found that TCM constitutions have influence on the repair ability of patients with FHN.^[9]^ It founds that the blood-stasis constitution has a strong repair capacity while the yang-deficiency constitution has a poor repair capacity and has a tendency to collapse.^[9]^ Another study has found that people with Yang-deficiency constitution, damp-heat constitution, and blood-stasis constitution are more likely to be diagnosed with FHN.^[10]^

Taohong Siwu decoction (TSD) is a famous Chinese herbal medicine prescription for regulating the menstrual function, which was first recorded in a Chinese ancient medical book called *the Golden mirror of medicine* written by imperial physician Qian Wu in 1742 AD.^[11]^ TSD is composed of Danggui (*Angelica sinensis*), Shudihuang (prepared rehmannia root), Chuanxiong (*Ligusticum wallichii*), Baishao (radix paeoniae alba), Taoren (peach kernel), Honghua (*Carthamus tinctorious*). TSD is used to promote the blood flow and prevent the blood stasis from happening.^[12,13]^ It also has been widely used in the treatment of osteoarthritis.^[14]^

According to the research of QI Zhen-xi, TSD is thought to be a therapy of glucocorticoid-induced ischemic necrosis of femoral head.^[13]^

However, we find that few systematic reviews are available. Hence, the aim of this protocol is to assess the effectiveness and safety of TSD for FHN according to current studies.

## 2 Methods

### 2.1 Study registration

PROSPERO CRD42019129771 is where the protocol being registered. This Systematic Reviews of Interventions follows the guidelines summarized in the Cochrane Handbook, while the preferred reporting items for systematic reviews and meta-analysis protocol is for protocol.

### 2.2 Inclusion criteria

#### 2.2.1 Types of included studies

For study inclusion, randomized controlled trials, which have assessed any types of TSD for the treatment of FHN, will be included. The randomized controlled trials time, geographical area, or language of publication will be unlimited.

#### 2.2.2 Types of participants

The participants will contain patients who were suffering from FHN. There will be no limits on other characteristics or factors, like the sex, age, and symptom severity.

#### 2.2.3 Types of interventions

Experimental group intervention contains control group intervention and TSD, which is taken orally. To avoid the influence of the dosage forms, we only take decoctions into consideration. Control group intervention could be placebo, blank control, conventional western medicine (such as Alendronate Sodium Tablets), or psychological intervention. If combined treatment of traditional Chinese and western medicine is given, both the treatment of western medicine in the control group and the experimental group should be coherent.

#### 2.2.4 Types of outcomes

##### 2.2.4.1 Primary outcomes

The primary outcome was the average pain on movement and function of hip. It was assessed by a 10-point visual analog scale.

##### 2.2.4.2 Secondary outcomes

The Western Ontario and McMaster Universities Osteoarthritis Index is the secondary outcome mainly indicator.

### 2.3 Data sources

#### 2.3.1 Electronic searches

Eight electronic databases, including the Cochrane Central Register of Controlled Trials, Web of Science, PubMed, EMBASE, China National Knowledge Infrastructure, Chinese Biomedical Literature Database, Technology Periodical database (Chinese Scientific Journal Database), and Wanfang Database. The time which will be searched is from the build-up time of respective databases to January 2020. There was no language restriction in this study. The detailed search strategy used in PubMed database will be presented in Supplemental Digital Content (Appendix S1).

#### 2.3.2 Other sources

Meanwhile, the relevant studies published on journals, along with their references, will be manually reviewed and retrieved as the supplementary literature.

### 2.4 Study selection

**Figure 1:**
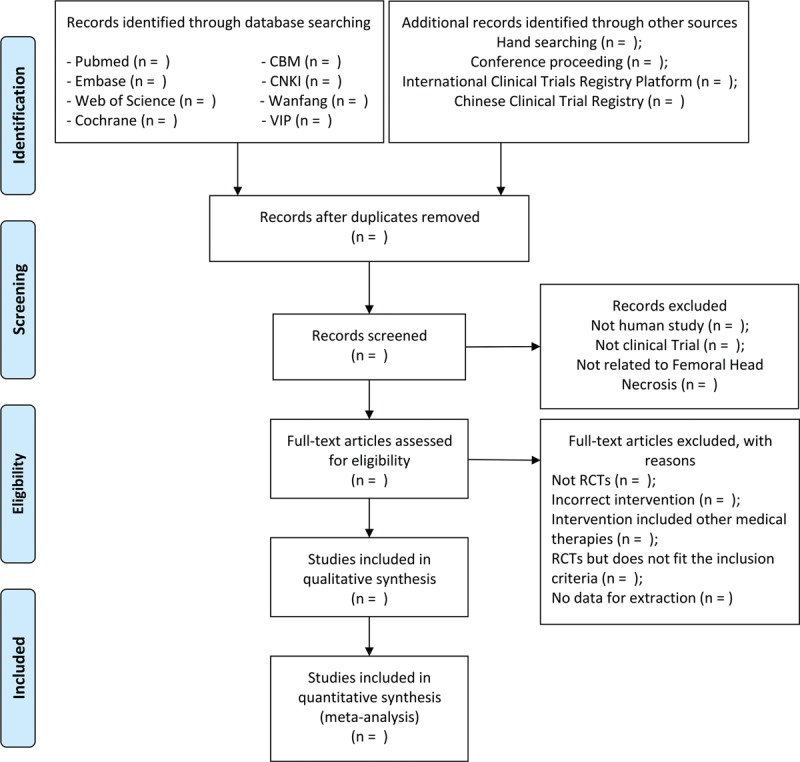
PRISMA flow chart of study selection process. PRISMA = preferred reporting items for systematic review and meta-analysis.

The referencing management software EndnoteX9 helps to cope with the results. Two researchers will independently conduct and cross-check the literature screening. First, by reading the titles of the references, they will rule out the obviously irrelevant literature. Then the abstract and full text will support us to determine if the studies should be reserved or not. Lastly, the inconsistencies will be resolved by another study member, who will also check the final inclusion of literature. The process of selection is revealed in a preferred reporting items for systematic reviews and meta-analysis flow diagram (Fig. 1).

### 2.5 Data collection and management

Data will be extracted into a pre-prepared form developed in accordance with Cochrane Handbook guidelines. They will be collected from the included trials by 2 authors (YL and SJ). Controversies will be resolved by consultation with an arbiter (GC). The extracted data will include publication time, the author name(s), sample size, age and sex of participants, intervention (regimen), control (regimen), visual analog scale, Western Ontario and McMaster Universities Osteoarthritis Index, other outcomes, and adverse effects.

### 2.6 Dealing with missing data

In consideration of missing or insuffient data, reviewers will contact the corresponding author for clarification when encountering this situation. Meanwhile, the potential impact about it will be discussed.

### 2.7 Assessment of risk of bias in included studies

Two independent researchers (PZ and PS) will appraise the risk of bias, according to the risk of bias tool in Cochrane Handbook. Consulting with the third author (GC) is necessary in case of any discrepancies coming up. Bias will be divided into 7 domains: selection bias, selection bias, performance bias, detection bias, detection bias, detection bias, and other bias. The evaluation results of the risks will be classified as low, uncertain, or high.

### 2.8 Measures of treatment effect

As continuity variables, the odds ratio value and 95% confidence interval indicate the effect, whereas the dichotomous data is appraised by relative risk.

### 2.9 Assessment of heterogeneity

We will apply random models to accomplish the analysis. Chi-squared and *I*-squared tests will help to appraise the heterogeneity among the studies. The case that *I*^*2*^ value is ≥50% will be interpreted as significant statistic heterogeneity, and vice versa. As the case occurs, appropriate subgroup analyses will be done to dig out the possible reason.

### 2.10 Detection of the likelihood of publication bias

Through using the Egger test, publication bias will be appraised. Funnel plots will be applied to detect potential reporting biases (at least 10 trials). Yet, since funnel plot asymmetries and publication biases have differences undoubtedly, we will try to find out the reasons for the asymmetry when it exists.

### 2.11 Data synthesis

The meta-analysis will be carried out with the software RevMan V.5.3. The data will be pooled as a mean difference or a risk ratio with 95% confidence intervals for continuous outcomes or the dichotomous. When heterogeneity is considered significant, the sensitivity or subgroup analysis will be carried out to explored the causes. When it mentions the data is inadequate for quantitative analysis, the conclusion will only show the evidence.

### 2.12 Subgroup analysis

The subgroup analysis will be performed based on different kinds of FHN, the patient's age, sex, and treatment period if a significant heterogeneity is presented in the included trials.

### 2.13 Sensitivity analysis

Sensitivity analysis will help to determine the effects of trial risk of bias on important outcomes. It will be conducted to verify the robustness of our primarily given assumptions. The principal decision points concluded the methodological quality, sample size, and the effect of missing data. With the low-quality literature and studies of small sample size ruled out, the meta-analysis will be conducted to assess whether these factors affect the results.

### 2.14 Quality of evidence

To confirm the quality of evidence for the outcomes, the grading of recommendations assessment, development, and evaluation will be utilized. It will be assorted into 4 levels as high, moderate, low, and very low levels. Flaws in risk of bias, indirectness, imprecision, inconsistency, and publication bias will be checked.

### 2.15 Ethics and dissemination

The results of this systematic review will offer implications of the use of TSD treatment for FHN. Given that the article will use published data instead of individual patient data, ethical board review and approval are not necessary. The findings of this review will be spread by being published in a peer-reviewed journal and disseminated in conference presentations.

## 3 Discussion

Femur head necrosis, a common orthopedic disease, has the therapy difficulty and high morbidity rate. Without complete cure having been established, FHN is a medical problem and a potentially crippling disease which seriously affects people's life.^[15,16]^ For younger patients, especially adolescents, surgical interventions of making hip joint preserved are more suitable. However, osteonecrosis is a progressive disease, hence it is vital to slow or reverse its progression.^[17]^ In this respect, the role of TCM is increasingly valued. TSD, a classic formula which can activate blood and resolve stasis, is a valid prescription to promote blood circulation and improve the microcirculation of femur head necrosis.

To date, there has been no systematic review on the effect of TSD for femur head necrosis. Hence we set about providing recent evidence related to the efficacy and safety of TSD in the treatment of femur head necrosis through this meta-analysis. Besides, we anticipate finding predictors of treatment and detecting more effective dosage and composition of TSD through subgroup analysis. It is our hope that the results of our study will provide guidance for clinicians and patients.

Nevertheless, our study, up to a point, is deficient due to the factors below. In the first place, articles written in other languages rather than English or Chinese will be left out. Although it seemingly brings about the loss of information, it is inappreciable by reason that the number of the researches is limited. In the second place, some studies might be of poor quality without mentioning the specific performance of blinding and randomization, for which the risk of bias will be applied for evaluation. In the last place, there may be selective reported outcomes, so we will gingerly abide by the rules of systematic review to eliminate unexpected studies.

## Author contributions

**Methodology:** Guoming Chen, Yaying Xie.

**Writing – original draft:** Guoming Chen, Yaying Xie.

**Writing – review & editing:** Guoming Chen.

Chi Zhou orcid: 0000-0003-1905-9494.

## Supplementary Material

Supplemental Digital Content
