# Supplementary material for: Taohong Siwu decoction for femoral head necrosis: A protocol for systematic review
Source: Medicine (Baltimore). 2020 Mar 27;99(13):e19368. doi: 10.1097/MD.0000000000019368 (PMC7220747; doi:10.1097/MD.0000000000019368)
Supplement: Supplemental Digital Content [file medi-99-e19368-s001.docx]

**Supplemental Digital Content (Appendix S1)**

**Search strategy used in PubMed database**

#1 Femoral Head Necrosis OR Femur Head Necroses OR Head Necrosis, Femur OR Necrosis, Femur Head OR Aseptic Necrosis of Femur Head OR Necrosis, Aseptic, of Femur Head OR Necrosis, Avascular, of Femur Head OR Ischemic Necrosis Of Femoral Head OR Femoral Head, Avascular Necrosis Of OR Avascular Necrosis Of Femoral Head, Primary OR Avascular Necrosis of Femur Head

#2 Taohong Siwu Decoction OR Tao Hong Si Wu Decoction OR THSW Decoction Taohong-Siwu Decoction OR Taohong Siwu Tang OR Tao Hong Si Wu Tang OR THSW Tang OR Taohong-Siwu Tang

#3 "randomized controlled trial"[pt] OR "controlled clinical trial"[pt] OR randomized[tiab] OR placebo[tiab] OR "drug therapy"[sh] OR randomly[tiab] OR trial[tiab] OR groups[tiab]

#1 AND #2 AND #3
